# Accelerated prefrontal intermittent theta-burst stimulation in Huntington’s disease: a within-subject study of domain-specific behavioral and event-related potential changes

**DOI:** 10.3389/fnhum.2026.1891708

**Published:** 2026-07-16

**Authors:** Marianna Delussi, Emmanuella Ladisa, Elena Ammendola, Giulia Paparella, Chiara Abbatantuono, Giusy Tancredi, Giulia Paparella, Elvira Brattico, Marina de Tommaso

**Affiliations:** 1 Department of Education, Psychology, Communication (For.Psi.Com.), University of Bari Aldo Moro (IT), Bari, Italy; 2 Neurophysiopathology Unit, Department of Translational Biomedicine and Neuroscience (DiBraiN), University of Bari Aldo Moro (IT), Bari, Italy; 3 University Niccolò Cusano, Rome, Italy; 4 IRCCS Neuromed, Pozzilli, Italy

**Keywords:** dorsolateral prefrontal cortex, event-related potentials, frontostriatal networks, Huntington’s disease, intermittent theta burst stimulation, neuromodulation, proof-of-concept study, transcranial magnetic stimulation

## Abstract

**Background:**

Huntington’s disease is a rare neurodegenerative movement disorder characterized by early disruption of frontostriatal systems, affecting motor, cognitive, and affective domains. Non-invasive brain stimulation targeting prefrontal networks may offer a means to modulate these distributed systems, although controlled evidence in Huntington’s disease remains limited, particularly for accelerated stimulation protocols.

**Objective:**

To investigate whether accelerated intermittent theta-burst stimulation (iTBS) applied to the dorsolateral prefrontal cortex is associated with behavioral, motor and electrophysiological changes reflecting modulation of prefrontal network function in early-stage Huntington’s disease.

**Methods:**

Ten patients with genetically confirmed Huntington’s disease participated in a within-subject, fixed-order longitudinal study, which included a sham exposure phase followed by active stimulation. Assessments were conducted at baseline (T0), after sham stimulation (T1), after active accelerated iTBS (T2), and at 60-day follow-up (T3). Clinical scales for motor impairment, behavioral measures targeting executive, affective, and social-cognitive domains were combined with event-related potentials (ERPs) recorded during cognitive and emotional Stroop tasks. The fixed-order design was chosen to minimize potential carry-over effects associated with accelerated stimulation protocols.

**Results:**

No significant behavioral or electrophysiological changes were observed during the sham exposure phase. In contrast, active iTBS was associated with domain-specific behavioral changes, particularly in affective, executive, and social-cognitive domains, accompanied by changes in event-related potential activity, particularly within delayed N200-related responses during emotional interference. Effects were domain-specific and were not associated with normalization of electrophysiological latency profiles. Motor scales were not modified by either sham or real stimulation.

**Conclusion:**

Accelerated prefrontal iTBS was associated with behavioral and electrophysiological changes following active stimulation of prefrontal network in Huntington’s disease. These findings support the feasibility of targeting distributed non-motor circuits in HD and designs accounting for cumulative and time-dependent effects of stimulation in early-phase neuromodulation studies.

## Highlights

- Active accelerated iTBS was associated with selective changes in executive, affective, and social-cognitive domains, with no comparable effects during sham stimulation in Huntington Disease.
- Electrophysiological modulation of frontal ERP markers was observed following active stimulation.
- Findings suggest that accelerated stimulation may amplify residual network responsiveness in Huntington Disease, with behavioral effects emerging selectively.

## 1 Introduction

Huntington’s disease (HD) is a rare autosomal-dominant neurodegenerative disorder caused by a CAG trinucleotide repeat expansion in the HTT gene, leading to progressive motor impairment, psychiatric symptoms, and cognitive decline (Ross and Tabrizi, 2011). Cognitive and affective disturbances emerge early in the disease course and contribute substantially to functional disability and reduced quality of life, even in the absence of severe motor involvement.

In addition to executive deficits, HD is characterized by impairments in social cognition, including emotion recognition, perspective-taking, and empathy, frequently accompanied by apathy and depressive symptoms (Larsen et al., 2016; Hendel et al., 2023). Disruption of executive–affective interactions is thought to contribute to these manifestations, reflecting impaired prefrontal regulation of emotional processing (Crocker and Heller, 2010). Such deficits are detectable in early disease stages and are associated with reduced social functioning and increased caregiver burden (Allain et al., 2011; Snowden et al., 2008).

Among cortical regions, the dorsolateral prefrontal cortex (DLPFC) is particularly vulnerable in HD, showing early structural and functional alterations (Rosas et al., 2002). The DLPFC plays a central role in executive control, motivational regulation, and the integration of cognitive and socio-emotional information (Miller and Cohen, 2001). It represents a key cortical node within frontostriatal circuits, which are critically affected in Huntington’s disease (Tabrizi et al., 2009). Non-invasive brain stimulation is thought to modulate cortical excitability and distributed network activity rather than isolated cortical targets (Pascual-Leone et al., 2000; Stagg and Rothwell, 2013; Chen et al., 2024). Repetitive TMS (rTMS) and intermittent theta-burst stimulation (iTBS) applied over the left DLPFC have been associated with modulation of executive and affective functions in both healthy individuals and clinical populations (Zhang et al., 2022; Lee et al., 2024). Increasing attention has recently been directed toward accelerated stimulation schedules, in which multiple daily sessions are delivered to enhance cumulative network engagement, although evidence in neurodegenerative disorders remains limited (Pagali et al., 2024).

In HD the potential benefits of DLPFC-targeted rTMS for depressive symptoms is mainly derived from case reports or small series (Jose et al., 2023; Gramegna et al., 2023; Schrader et al., 2021; Boyadjian et al., 2022). Systematic reviews consistently emphasize methodological heterogeneity, limited use of sham-controlled designs, and a lack of controlled studies targeting cognitive and socio-affective outcomes in HD (Gramegna et al., 2023; Schrader et al., 2021; Clemente et al., 2025).

In parallel, cognitive and emotional impairment in prefrontal functions in HD is detected early with the Stroop test, which has a robust neurophysiological correlate (Migliore et al., 2024; Folstein and Van Petten, 2008; Ergen et al., 2014; Thomas et al., 2007). These measures provide time-sensitive, non-invasive indices of prefrontal network dynamics and may offer complementary insight into neuromodulation-related changes beyond behavioral outcomes alone.

Theta burst stimulation (TBS) represents a specialized variant of repetitive transcranial magnetic stimulation (rTMS), a non-invasive neuromodulation technique widely employed to influence brain circuits implicated in neurological and psychiatric conditions. Compared with standard rTMS approaches, TBS requires considerably shorter treatment sessions and can achieve therapeutic effects using lower stimulation intensities, while preserving comparable safety and effectiveness. More recently, accelerated TBS paradigms have been developed to administer multiple stimulation sessions within a condensed timeframe, increasing the cumulative dose of stimulation and potentially enhancing the speed and magnitude of clinical improvement. Current evidence supports the safety, efficacy, and sustained therapeutic benefits of these accelerated protocols relative to conventional TBS approaches (Cole et al., 2024).

To date, controlled evidence examining the effects of intermittent theta-burst stimulation (iTBS) on cognitive–affective domains in Huntington’s disease, together with concurrent electrophysiological modulation, remains limited. Within the context of a neurodegenerative movement disorder characterized by early disruption of frontostriatal systems, the dorsolateral prefrontal cortex represents a key cortical node within distributed networks supporting executive control, affect regulation, and goal-directed behavior.

The present pilot study therefore aimed to investigate whether an accelerated iTBS protocol targeting the left dorsolateral prefrontal cortex is associated with domain-specific behavioral and neurophysiological changes in individuals with early-stage Huntington’s disease. This could be particularly relevant in a disease lacking specific disease modifying therapies and treatments with proven effects on cognitive functions (Bachoud-Lévi et al., 2019).

To check for prefrontal cognitive and emotional functions, we used EEG activity related to cognitive and emotional Stroop test paradigm (Folstein and Van Petten, 2008; Ergen et al., 2014; Thomas et al., 2007). To address methodological challenges inherent to potential carry over effect of accelerated theta burst stimulation paradigms, a within-subject, fixed-order design including a sham exposure phase followed by active stimulation was adopted. Given the exploratory nature of the study and the rarity of Huntington’s disease, this approach aligns with early-phase neuromodulation research, where within-subject sensitivity and feasibility considerations may take precedence over full randomization.

## 2 Methods

### 2.1 Participants

#### 2.1.1 Subjects

Ten patients with genetically confirmed HD were consecutively recruited from the Apulian Referral Center for Huntington’s Disease between January 2024 and July 2025. Inclusion criteria were: age >18 years, CAG repeat length ≥40, Total Functional Capacity (TFC) score ≥7, and stable pharmacological treatment for at least 8 weeks before enrollment. Exclusion criteria included a history of seizures, metallic implants, pacemakers, or other contraindications to TMS, in accordance with international safety guidelines (Cole et al., 2024).

#### 2.1.2 Study design

**Figure 1:**
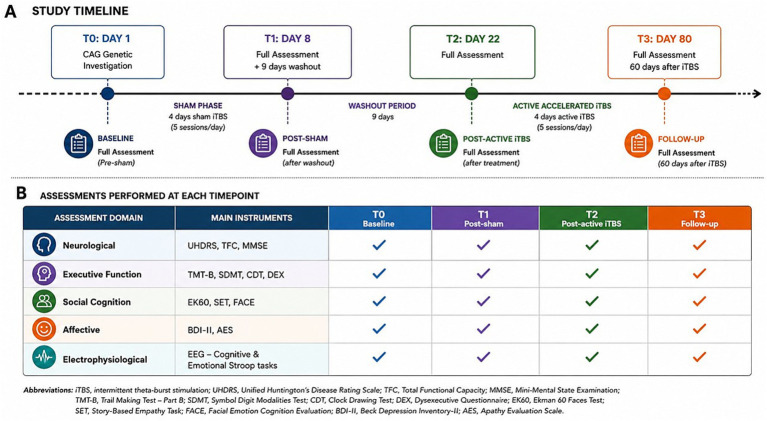
Overview_ single-blind, sham-controlled, fixed-order within-subject study design and stimulation timeline, including baseline (T0), post-sham (T1), post-active iTBS (T2), and 60-day follow-up (T3) assessments. **(A)** Study Timeline, **(B)** Assessments performed at each Timepoint.

Assessments were conducted at four time points T0, T1 (3 days after sham stimulation), T2 (3 days after active iTBS), and T3 (60 days after active stimulation). This pilot study adopted a within-subject, fixed-order longitudinal design including a sham exposure phase followed by active stimulation, with repeated assessments across four timepoints (T0–T3) (Figure 1).

The fixed-order structure (sham → active) was intentionally selected based on neurobiological considerations related to accelerated stimulation protocols. Accelerated intermittent theta-burst stimulation (iTBS), delivered in multiple daily sessions, is thought to induce cumulative and potentially non-linear plasticity-like effects that may persist beyond conventional washout intervals (Rossi et al., 2009), suggesting that conventional washout intervals may be insufficient to fully eliminate carry-over effects. In this context, counterbalanced or randomized crossover designs may introduce carry-over effects that are difficult to quantify and may confound condition-specific interpretation.

Participants were blinded to the stimulation condition and were informed that the sham and real sessions would be conducted in a random, unpredictable manner.

Neuropsychological tests and EEG recordings were anonymized to allow blind evaluation of data.

The Ethical Committee of Bari Policlinico General Hospital approved the study.

#### 2.1.3 Neurological assessment

Motor manifestations and disease stage were assessed using the Diagnostic Confidence Level (DCL) and Total Motor Score (TMS) of the Unified Huntington’s Disease Rating Scale (UHDRS) (Huntington Study Group, 1996; Hogarth et al., 2005). Functional autonomy and disease severity were evaluated using the Total Functional Capacity (TFC) scale (Shoulson and Fahn, 1979). Global cognitive status was screened using the Mini-Mental State Examination (MMSE) (Folstein et al., 1975).

#### 2.1.4 Genetic investigation

Genetic diagnosis was confirmed by polymerase chain reaction amplification and capillary electrophoresis analysis of CAG trinucleotide repeats in the HTT gene, using DNA extracted from peripheral blood lymphocytes. A threshold of ≥40 CAG repeats was considered indicative of full penetrance HD, in line with established molecular criteria (Ross et al., 2014).

#### 2.1.5 Neuropsychological assessment

Executive functions were assessed using the Trail Making Test–Part B (TMT-B) (Reitan, 1992), Symbol Digit Modalities Test (SDMT) (Smith, 1982), Clock Drawing Test (CDT) (Freedman et al., 1994), and the Dysexecutive Questionnaire (DEX) (Wilson et al., 1996). Social cognition was evaluated using the Ekman 60 Faces Test and the Faces Test (FACE) (Young et al., 2002; Terruzzi et al., 2023), as well as the Story-based Empathy Task (SET) (Adenzato et al., 2010). Depressive symptoms and apathy were assessed using the Beck Depression Inventory-II (BDI-II) and the Apathy Evaluation Scale (AES), respectively. All instruments are validated for use in HD populations and are sensitive to frontostriatal dysfunction (Caillaud et al., 2024).

#### 2.1.6 Stroop task: cognitive session

The stimuli were displayed individually on a black-screen monitor. During the test, a list of words (green, blue, and red) was presented in a random order. However, the color of the words could match the presented word or not (e.g., the word “blue” in blue ink or the word “blue” in green ink). Subjects were then asked to consider the color of the ink and ignore the word’s semantic meaning (e.g., answer “green” instead of “blue,” as in the previous example) by quickly pressing the corresponding color-coded key on a keypad with three colored keys (red, green, and blue). A total of 60 stimuli were administered, which were displayed on the screen for two seconds, each five seconds apart. The stimuli consisted of the words “blue,” “red,” and “green” and were presented in randomized order as follows: 10 congruent “blue” (the word “blue” was colored blue); 10 congruent “red”; 10 congruent “green”; 10 incongruent “blue” (five colored red and five colored green); 10 incongruent “red” (five colored blue and five colored green); and 10 incongruent “green” (five colored red and five colored blue) (Heidlmayr et al., 2020). Correct (c), wrong (w), and missing (m) responses were considered.

#### 2.1.7 Stroop task: emotional session

A series of words in Italian, selected for their potential to attract attention and characterized by different emotional valences (positive, negative, neutral), were shown in random order. The words were presented in one of three different colors: green, blue, red. Participants were asked to recognize only the color of the ink and to ignore the semantic meaning of the word and respond as quickly as possible by pressing the corresponding color coded key on a keypad with three colored keys (red, green, blue) A total of 48 stimuli were presented, each of which was displayed for two seconds, with an eight-second interval between one presentation and the next. The stimuli included 16 words with positive valence, 16 words with negative valence and 16 words with a neutral value. The four presentation colors (green, red, blue) were used in a counterbalanced distribution, with each color appearing 12 times at random (Gootjes et al., 2011). Correct (c), wrong (w) and missing (m) responses were considered. Reaction time distributions were inspected to verify the absence of extreme or non-physiological values; no trials met criteria for exclusion.

#### 2.1.8 iTBS procedure

Intermittent theta-burst stimulation was delivered using an STM9000 stimulator (EB Neuro) equipped with a focal figure-of-eight (butterfly-shaped) coil. Stimulation was applied by positioning the coil tangentially to the scalp over the left DLPFC, resulting in a perpendicular induced current relative to the cortical surface. Neuronavigation (NetBrain Neuronavigator) was performed at baseline to localize the left DLPFC and repeated before each daily stimulation session to ensure reproducible coil positioning. Resting motor threshold (RMT) was defined as the minimum stimulus intensity eliciting a motor evoked potential (MEP) of at least 50 microvolts and recorded at the right abductor pollicis brevis muscle in at least 7 of 10 trials. MEP was obtained by using a circular coil. Stimulation intensity was set at 110% of individual RMT. Target engagement was ensured through repeated verification of coil positioning before each stimulation session.

The canonical iTBS protocol was applied, consisting of bursts of three pulses at 50 Hz repeated at 5 Hz, delivered in 2-s trains every 8 s. Participants received four consecutive days of stimulation, with five sessions per day, each delivering 1,620 pulses. This resulted in a daily dose of 6,480 pulses and a cumulative total of 32,400 pulses, which enabled efficient stimulation in a short time and reduced the burden for patients and caregivers, in accordance with several existing protocols (Cole et al., 2024). Sessions were separated by 15-min intervals. This interval was the most frequently used, particularly in studies on depression, including larger series (Cole et al., 2024).

Sham stimulation was delivered using the integrated sham mode of the stimulation system, designed to reproduce the acoustic characteristics of active stimulation without inducing effective cortical modulation (Loo et al., 2006). Identical coil positioning and procedural parameters were maintained across conditions to preserve perceptual similarity (Cole et al., 2024; Smith et al., 2019).

#### 2.1.9 EEG recording technique

EEG activity was recorded using the Micromed Brain Quick system (Natus, Middleton, WI, USA) with a 61-electrode high-density cap arranged according to the extended international 10–20 system. A biauricular reference was used, and the ground electrode was placed on the right forearm. Eye movements were monitored using electrodes placed at the outer canthi. Signals were sampled at 256 Hz with a bandpass filter of 0.1–70 Hz and a 50-Hz notch filter. EEG was recorded during performance of both a cognitive and an emotional Stroop task.

#### 2.1.10 EEG preprocessing

EEG data were processed using EEGLAB (version R2023b) running on MATLAB (MathWorks Inc.). Continuous data were filtered between 1 and 30 Hz. Artifact Subspace Reconstruction was applied to detect and correct contaminated data segments and channels, followed by interpolation and re-referencing to the common average. Independent component analysis (ICA) was computed using the extended infomax algorithm implemented in EEGLAB. Artifactual components were automatically classified and rejected using the Multiple Artifact Rejection Algorithm (MARA). EEG data were then segmented into epochs from −0.1 to 1 s relative to stimulus onset and baseline-corrected. Final component rejection was visually inspected to ensure physiological plausibility and avoid overcorrection.

#### 2.1.11 EEG-event-related potential analysis

ERP analyses were conducted during cognitive and emotional Stroop tasks. Task parameters followed established paradigms (Folstein and Van Petten, 2008; Ergen et al., 2014). ERP components were identified according to established temporal criteria, with analyses focusing on N200, N450 (Folstein and Van Petten, 2008; Ergen et al., 2014). Epochs were averaged separately by condition and time point. Latency and amplitude measures were extracted at frontocentral electrodes, and topographical analyses were performed across the 61-electrode montage using Letswave (version 7).

To assess baseline electrophysiological performance, ERP responses of the Huntington’s disease (HD) group at T0 were compared with those of a group of 10 age- and sex-matched healthy control subjects (HD mean age = 48.3 years; controls = 50.0 years). Control participants were recruited through institutional advertising and underwent clinical screening to exclude first-degree familial Huntington’s disease history, neurological disorders, or relevant systemic medical conditions. Latencies of main waves were computed on averaged frontocentral channels. For the cognitive Stroop task, ERP data were analyzed separately for congruent (e.g., the word “red” presented in red ink) and incongruent (e.g., the word “red” presented in green ink) stimuli. ERP epochs were averaged separately for each condition in both HD patients and control participants at T0, T1, T2, and T3. For the cognitive Stroop task, 30 artifact-free epochs were averaged per condition (congruent and incongruent). For the emotional Stroop task, 16 artifact-free epochs were averaged for each emotional valence (positive, negative, and neutral).

### 2.2 Statistical analysis

A predefined statistical analysis plan was implemented to evaluate longitudinal changes in motor, cognitive, affective, and neurophysiological outcomes following intermittent theta-burst stimulation (iTBS) across four assessment timepoints (T0, T1, T2, T3), while accounting for within-subject variability and potential clinical predictors of response.

#### 2.2.1 Behavioral and cognitive analyses

Normality of outcome distributions was assessed using the Shapiro–Wilk test. Based on distributional properties, either parametric (paired-sample *t*-tests) or non-parametric (Wilcoxon signed-rank tests) comparisons were applied for pairwise contrasts between consecutive timepoints (T1–T0: sham; T2–T1: active stimulation; T3–T2: follow-up). These analyses were conducted to characterize short-term and interval-specific changes across outcome domains. For each contrast, mean change (*Δ*), raw *p*-values, false discovery rate (FDR)–corrected *p*-values using the Benjamini–Hochberg procedure, effect sizes (Cohen’s dz. for parametric tests or rank-based r for non-parametric tests), and 95% confidence intervals were reported. These pairwise analyses were considered descriptive and hypothesis-generating. Given the fixed-order design, analyses focused on interval-specific contrasts (T1–T0 and T2–T1) to reduce temporal confounding and isolate stimulation-specific effects. The absence of significant affective changes during the sham interval (T1–T0), together with the temporal specificity of changes observed between T1 and T2, was interpreted as an internal control against simple time-dependent or practice-related effects. To provide a more robust assessment of longitudinal effects while accounting for inter-individual variability typical of HD (Tabrizi et al., 2009), linear mixed-effects models (LMMs) were fitted separately for each behavioral, executive, affective, and social-cognitive outcome. Time was modeled as a categorical fixed effect (reference level: T0), while age, baseline MMSE score, and CAG repeat length were included as covariates based on their established relevance to clinical progression in HD (Tabrizi et al., 2011; Paulsen et al., 2008). Subject identity was modeled as a random intercept to account for repeated measures within individuals. Fixed-effect estimates (*β*), standard errors, *p*-values, and 95% confidence intervals were reported. This modeling approach has been widely adopted in observational and interventional studies in Huntington’s disease (Paulsen et al., 2008). All statistical tests were two-tailed with a significance threshold set at *α* = 0.05. Multiple comparisons were controlled using FDR correction for pairwise tests, whereas unadjusted *p*-values were reported for LMMs, in line with current recommendations for small-N longitudinal designs (Luke, 2017). To explore potential predictors of treatment-related change, exploratory linear regression analyses were conducted using change scores (*Δ*) across three predefined intervals: sham (ΔT1–T0), active stimulation (ΔT2–T1), and follow-up (ΔT3–T2). Each regression model included age, baseline MMSE score, CAG repeat length, and the baseline value of the corresponding outcome variable as predictors.

#### 2.2.2 ERP statistical analysis

For ERP data, baseline group comparisons and longitudinal within-subject effects were assessed using repeated-measures analyses and paired-sample *t*-tests, as appropriate. To control for multiple comparisons across electrodes and timepoints, statistical significance was evaluated using cluster-based permutation procedures implemented in Letswave software. This approach was adopted to account for the spatially and temporally extended nature of ERP effects and to provide a conservative assessment of statistical significance. All statistical analyses were performed using Letswave software (version 7). Cluster-based statistical results were visualized using scalp topographic maps to illustrate the spatial distribution of significant effects across electrodes and time windows. For comparison of ERP latencies computed over averaged frontocentral electrodes between patients and controls and across the different stimulation conditions, we implemented, respectively, the Student’s te test and repeated measures ANOVA with pairwise comparisons.

Detailed quantitative results are reported in the Results section and Supplementary materials.

## 3 Results

### 3.1 Demographic and clinical variables

Baseline demographic and clinical characteristics of the 10 participants with Huntington’s disease (HD) are summarized in Table 1. The sample comprised 7F/3M, mean age of 44.9 ± 13.8 years and a mean education level of 14.0 ± 4.6 years. Baseline cognitive and functional measures were consistent with early-to-mid disease stages, as reflected by preserved global cognition (MMSE = 27.3 ± 1.8), functional capacity (TFC = 10.3 ± 2.3), and mild executive impairment (CDT = 2.3 ± 3.1). Descriptive statistics for all neuropsychological, affective, and functional outcomes across the four assessment timepoints (T0–T3) are reported in Table 2.

**Table 1:** Baseline demographic and clinical data (*N* = 10).

Subject	Gender	YY of illness	Education	Age	CAG	MMSE	TFC	CDT
P1	Female	2.0	18.0	36.0	49.0	28.0	11.0	3.0
P2	Female	1.0	13.0	37.0	53.0	25.0	7.0	7.0
P3	Female	0.0	17.0	28.0	48.0	30.0	13.0	0.0
P4	Male	7.0	5.0	71.0	41.0	27.0	10.0	0.0
P5	Male	5.0	8.0	78.0	39.0	26.0	7.0	4.0
P6	Female	2.0	17.0	53.0	42.0	27.0	13.0	0.0
P7	Male	5.0	13.0	43.0	41.0	26.0	10.0	3.0
P8	Male	2.0	8.0	55.0	43.0	26.0	10.0	0.0
P9	Male	0.0	13.0	30.0	42.0	25.0	11.0	5.0
P10	Female	4.0	17.0	52.0	42.0	28.0	12.0	0.0

**Table 2:** Longitudinal measures of all variables across time points.

Variable	T0	T1	T2	T3
UHDRS	20.3 ± 13.82	19.7 ± 13.56	19.1 ± 13.96	19.5 ± 13.79
DEX	36.9 ± 18.45	37.3 ± 19.41	31.0 ± 16.49	33.6 ± 17.18
AES	16.8 ± 6.6	17.6 ± 7.57	11.5 ± 7.04	10.4 ± 6.83
BDI II	19.5 ± 10.39	18.6 ± 9.38	12.5 ± 7.82	13.9 ± 8.52
SDMT	20.4 ± 12.68	19.3 ± 13.21	22.2 ± 12.63	20.5 ± 12.16
TRAIL-B	155.1 ± 57.35	156.1 ± 56.67	140.2 ± 52.64	140.2 ± 48.89
SET	12.5 ± 2.42	12.3 ± 2.31	12.8 ± 2.62	11.9 ± 2.28
FACE	22.1 ± 5.22	22.4 ± 5.93	26.4 ± 7.21	24.9 ± 6.42
EKMAN 60f	39.6 ± 8.62	36.8 ± 9.66	38.2 ± 9.35	37.6 ± 9.26
STROOP mean-RT	3485.47 ± 3171.27	3230.8 ± 3343.01	2974.7 ± 3095.78	2949.81 ± 2995.97
STROOP n.corr	51.7 ± 9.44	54.9 ± 4.36	58.0 ± 3.65	55.0 ± 4.92
STROOP n.wrong	3.7 ± 3.95	1.9 ± 2.28	1.2 ± 1.69	1.1 ± 1.45
STROOP n.miss	4.6 ± 7.0	2.7 ± 3.3	0.8 ± 2.2	3.9 ± 5.28
E-STROOP mean-RT	1722.96 ± 635.84	2483.51 ± 2644.14	2371.91 ± 2657.47	1496.91 ± 561.43
E-STROOP n. corr	43.8 ± 5.47	42.5 ± 7.55	44.5 ± 4.12	46.0 ± 1.05
E-STROOP n.wrong	0.8 ± 1.23	0.7 ± 0.95	0.7 ± 1.06	1.3 ± 1.25
E-STROOP n.miss	3.4 ± 4.81	4.2 ± 7.27	2.7 ± 4.42	0.8 ± 1.03

### 3.2 Behavioral and cognitive outcomes

**Figure 2:**
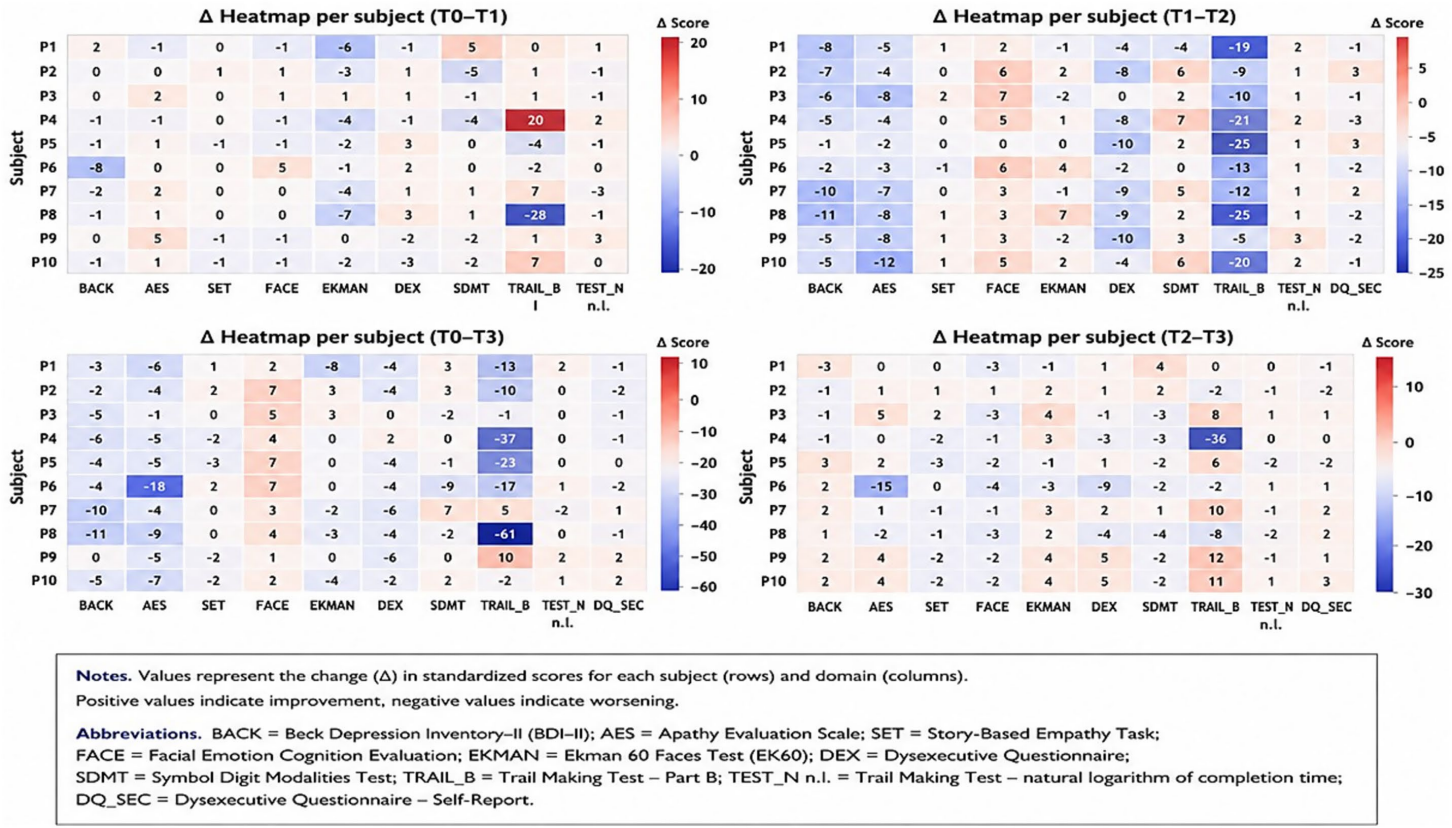
Heatmaps illustrate raw change scores (*Δ*) for each participant across outcome measures between consecutive time points (T1–T0, T2–T1, T3–T2) and across the overall interval (T3–T0). Color scale represents the magnitude and direction of change, highlighting inter-individual variability across cognitive, affective, and social-cognitive domains.

To facilitate visualization of longitudinal changes, *Δ*-heatmaps were generated for each temporal transition (T1–T0, T2–T1, T3–T2, and T0–T3), at both individual and group levels. These heatmaps display effect size magnitude and statistical significance across outcome domains and are presented in Figure 2. Motor symptoms, as measured by the UHDRS, remained stable during the task (Table 2).

Sham stimulation phase (T0–T1): Pairwise comparisons revealed no significant behavioral or cognitive changes following sham stimulation. Details of analysis are reported in Supplementary material. Consistent with these findings, *Δ*-heatmaps displayed in Figure 2 showed an absence of systematic effects across cognitive, affective, and social domains during the sham condition. All comparisons remained non-significant after false discovery rate (FDR) correction.

Active iTBS phase (T1–T2): In contrast, a distinct pattern of change emerged following active iTBS. Significant improvements were observed across multiple affective, executive, and social-cognitive measures. Depressive symptoms decreased markedly (BDI-II *Δ* = −6.1, *p* = 0.00058), accompanied by a significant reduction in apathy (AES *Δ* = −6.1, *p* = 0.00013). Executive dysfunction, as measured by the Dysexecutive Questionnaire, also improved (DEX Δ = −6.3, *p* = 0.00043). Social-cognitive performance increased significantly (FACE Δ = +4.0, *p* = 0.00024), with the largest effect observed in cognitive flexibility (TRAIL-B Δ = −15.9 s, *p* = 0.00005). Processing speed showed a modest but statistically significant improvement (SDMT *Δ* = +2.9, *p* = 0.022), although this effect did not survive FDR correction. The individual- and group-level *Δ*-heatmaps in Figure 2 confirmed the consistency of these improvements across participants, with the largest effect sizes observed in affective and executive domains.

Follow-up phase (T2–T3) and overall change (T0–T3): During the follow-up interval, partial attenuation of cognitive gains was observed, whereas affective improvements were largely preserved. Depression scores showed a small rebound (BDI-II *Δ* = +1.4, *p* = 0.013) but remained significantly improved relative to baseline. Social cognition declined moderately (FACE Δ = −1.5, *p* = 0.015). Executive outcomes were more variable, with a non-significant worsening on DEX (Δ = +2.6, *p* = 0.075) and a slight decline in processing speed (SDMT Δ = −1.7, *p* = 0.17). Across the full study period (T0–T3), sustained improvements were evident in affective domains (BDI-II Δ = −5.6, *p* = 0.00046; AES Δ = −6.4, *p* = 0.0019), alongside partial maintenance of gains in social cognition (FACE Δ = +2.8, *p* = 0.028) and executive functioning (DEX Δ = −3.3, *p* = 0.004). Full pairwise statistics are reported in Supplementary Table 2.

### 3.3 Linear mixed-effects models

To account for inter-individual variability and baseline clinical and genetic factors, linear mixed-effects models were fitted for each behavioral, executive, and social-cognitive outcome. After adjustment for age, baseline MMSE, and CAG repeat length, significant improvements at T2 relative to baseline were confirmed for depression, executive functioning (DEX), social cognition (FACE), and cognitive flexibility (TRAIL-B), with all *p*-values < 0.01. Processing speed showed a positive but non-significant trend. At the 60-day follow-up, improvements remained significant for depression, executive functioning and social cognition. Full model coefficients and confidence intervals are detailed in Supplementary Table 3.

### 3.4 Exploratory predictor analyses

Exploratory regression analyses were conducted to examine potential clinical predictors of change. Given the limited sample size, these analyses were considered hypothesis-generating and are reported in detail in the Supplementary Table 4.

### 3.5 Covariate effects

Across models, covariate effects aligned with known clinical features of HD. Older age was negatively associated with social-cognitive outcomes, while baseline MMSE strongly predicted executive and social-cognitive performance. CAG repeat length was inversely associated with apathy and showed trend-level associations with social-cognitive measures. Importantly, the main effect of Time remained significant after covariate adjustment, indicating that stimulation-related changes were not fully explained by baseline demographic, cognitive, or genetic factors (Supplementary Table 5).

### 3.6 ERP analysis

#### 3.6.1 Cognitive Stroop test

HD and control groups were matched for age and sex (HD mean age = 48.3 years; controls = 50.0 years). At baseline (T0), participants with HD exhibited a trend toward slower response times during the cognitive Stroop task compared with controls (HD = 2684.9 ms; controls = 1219.0 ms; *t*(9.2) = 1.90, *p* = 0.087), together with a trend toward fewer correct responses (HD = 51.7; controls = 58.0; *t*(10.3) = −2.03, *p* = 0.069) and a higher number of missed trials (HD = 4.6; controls = 0.4; *t*(9.7) = 1.88, *p* = 0.091). Incorrect responses were slightly more frequent in the HD group (HD = 3.7; controls = 1.6; *t*(9.5) = 1.37, *p* = 0.188), although this difference did not reach statistical significance (Supplementary Table 5).

**Figure 3:**
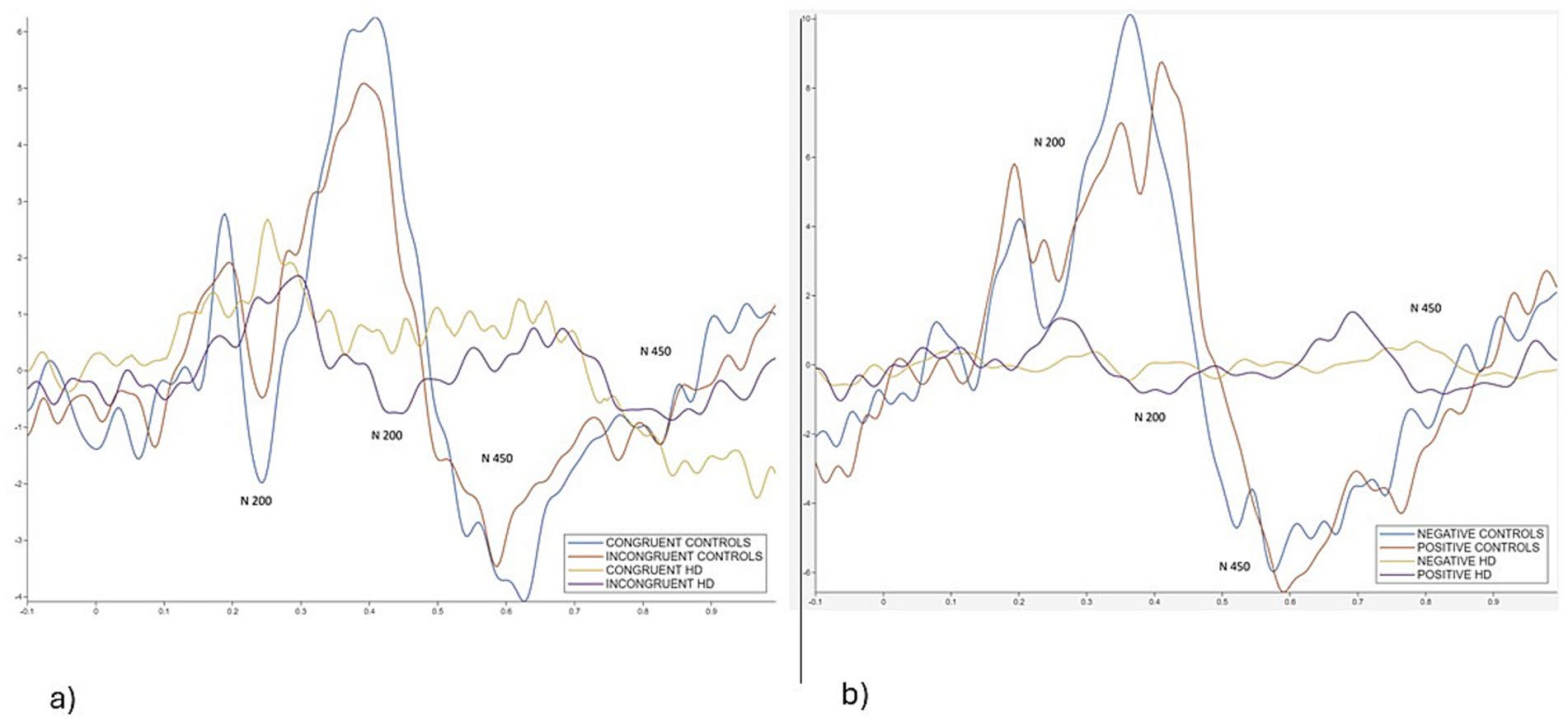
**(a)** Grand-average ERPs elicited during the cognitive Stroop task by congruent and incongruent stimuli in HD patients and healthy controls at baseline (T0), over frontocentral electrodes. **(b)** Grand-average ERPs elicited during the emotional Stroop task by negative, positive, and neutral stimuli in HD patients and healthy controls at baseline (T0), over frontocentral electrodes.

At baseline (T0), ERP responses during the cognitive Stroop task differed between HD patients and healthy controls. In the HD group, ERPs were characterized by prolonged component latencies, increased temporal dispersion, and reduced peak definition across both congruent and incongruent conditions (Figure 3a). On averaged frontocentral electrodes, negative components within the N200-related (150–350 ms) and N450-related (350–550 ms) time windows were markedly delayed also out of the time windows in HD compared with controls for both stimulus types. Due to pronounced latency variability in the patient group, point-by-point amplitude comparisons within fixed temporal windows were not considered reliable. Accordingly, peak-to-peak amplitude measures were used, revealing a significant reduction of N200 and N450 related activities in HD. Details of latency and amplitude statistical comparisons between HD and controls in regard to N200 and N450 amplitude are reported in Supplementary Table 6.

**Figure 4:**
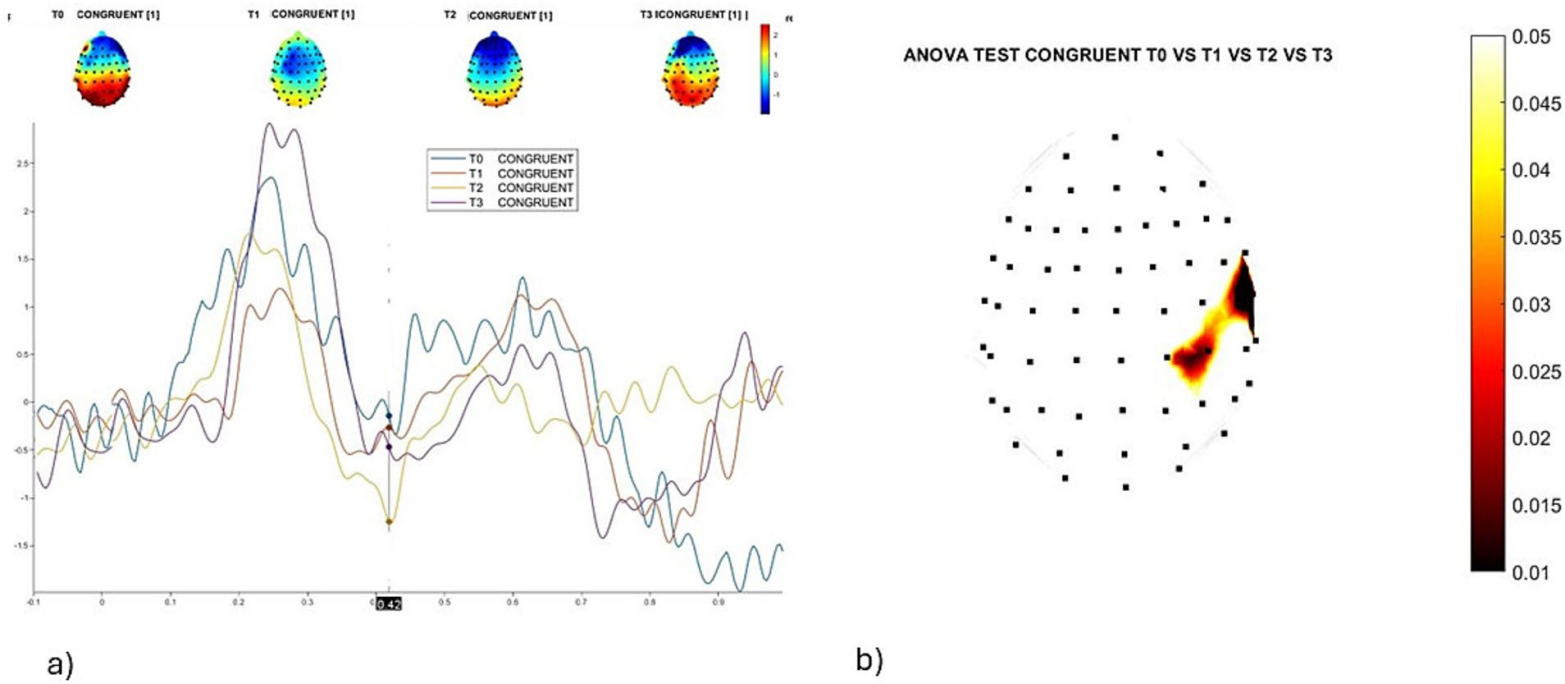
**(a)** Grand-average ERP waveforms (lower panels) and corresponding scalp topographies (upper panels) during the congruent condition of the cognitive Stroop task across time points. **(b)** Topographic representation of electrode-wise *p*-value distributions derived from repeated-measures ANOVA applied to cognitive Stroop ERP data across stimulation sessions.

Longitudinal analyses examined ERP modulation across sham stimulation (T1), active intermittent theta-burst stimulation (iTBS; T2), and follow-up (T3). No latency reduction was observed after sham and active stimulation. For congruent stimuli, ERP amplitude of the delayed N200 response did not change following sham stimulation. After active iTBS, a moderate increase in amplitude was observed over frontal regions, with partial persistence at follow-up (Figure 4a). Repeated-measures ANOVA indicated modulation of delayed N200-related activity over centro-parietal regions, although *post hoc* pairwise comparisons did not reach statistical significance (Figure 4b). A cluster-level effects for later ERP activity were observed across stimulation timepoints (Figure 4b).

**Figure 5:**
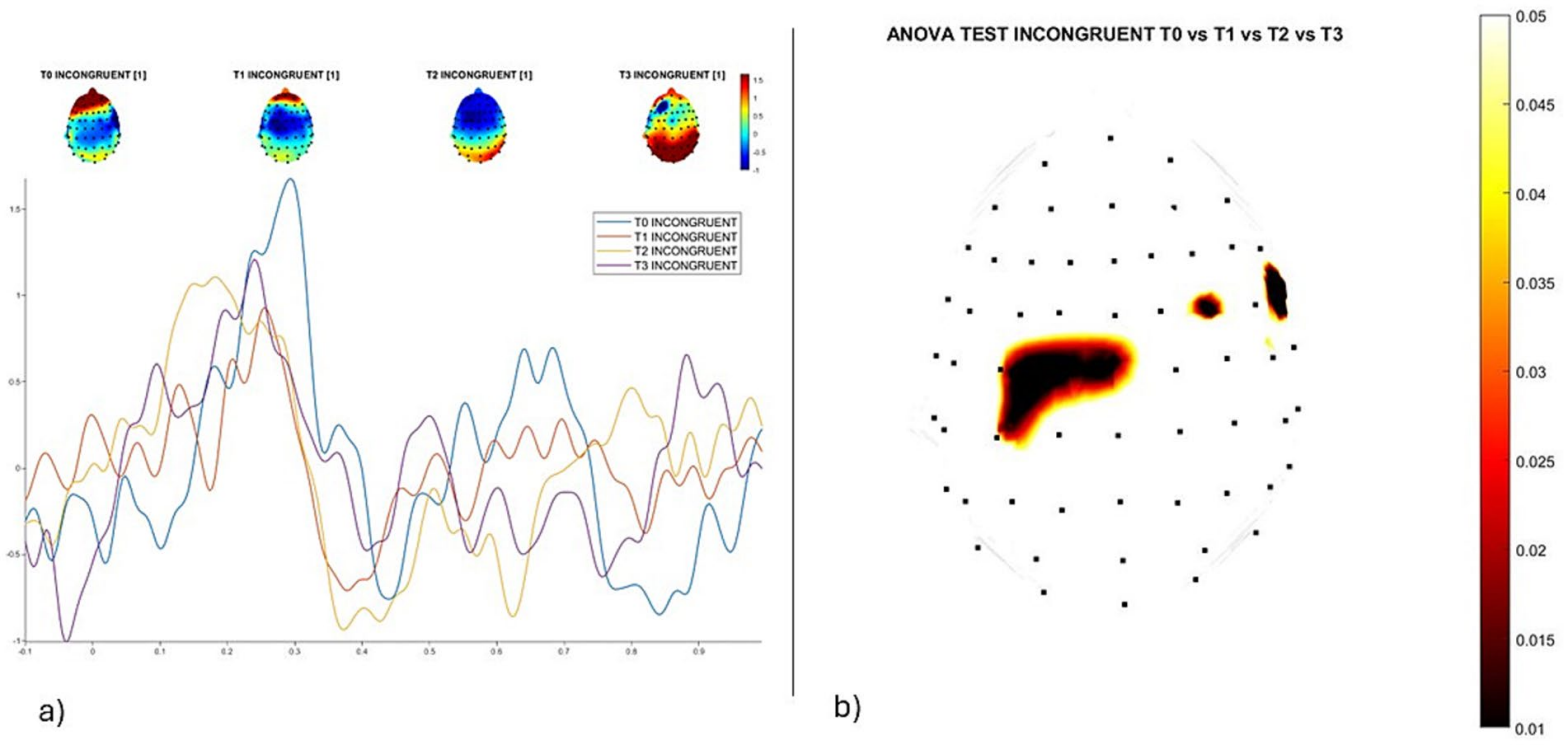
**(a)** Grand-average ERP waveforms (lower panels) and corresponding scalp topographies (upper panels) during the incongruent condition of the cognitive Stroop task across time points. **(b)** Topographical representation of clusters showing significant time point–related effects for incongruent stimuli during the cognitive Stroop task, identified using repeated-measures ANOVA with cluster-based permutation correction. Effects display a spatially distributed pattern across electrodes.

For the incongruent stimuli, a mild increase of delayed N200 amplitude was observed in T2, with a significant comparison of repeated measures ANOVA on the central electrodes (Figures 5a,b). Also in this case post hoc pairwise comparison was not significant.

Overall, ERP results from the cognitive Stroop task indicate altered temporal dynamics of conflict-related processing at baseline in HD patients and subtle, spatially distributed electrophysiological changes following active prefrontal neuromodulation.

#### 3.6.2 Emotional Stroop test

During the emotional Stroop task, HD patients exhibited longer reaction times compared with healthy controls (HD = 1722.5 ms; controls = 1058.8 ms; *t*(8.9) = 2.97, *p* = 0.011). The number of correct responses was slightly lower in the HD group, without reaching full statistical significance. Error rates were comparable between groups, whereas missed responses were more frequent in HD patients, showing a trend toward significance (Supplementary Table 5).

At baseline (T0), ERP responses elicited by emotional stimuli in the HD group differed from those of controls, showing prolonged latencies, increased temporal dispersion, and reduced peak definition, consistent with the pattern observed in the cognitive Stroop task (Figure 3b) Both positive and negative stimuli elicited delayed negative activity attributable to N200 and N450 responses. Latency measures computed on averaged frontocentral electrodes confirmed a significant temporal shift relative to controls (Supplementary Table 6).

**Figure 6:**
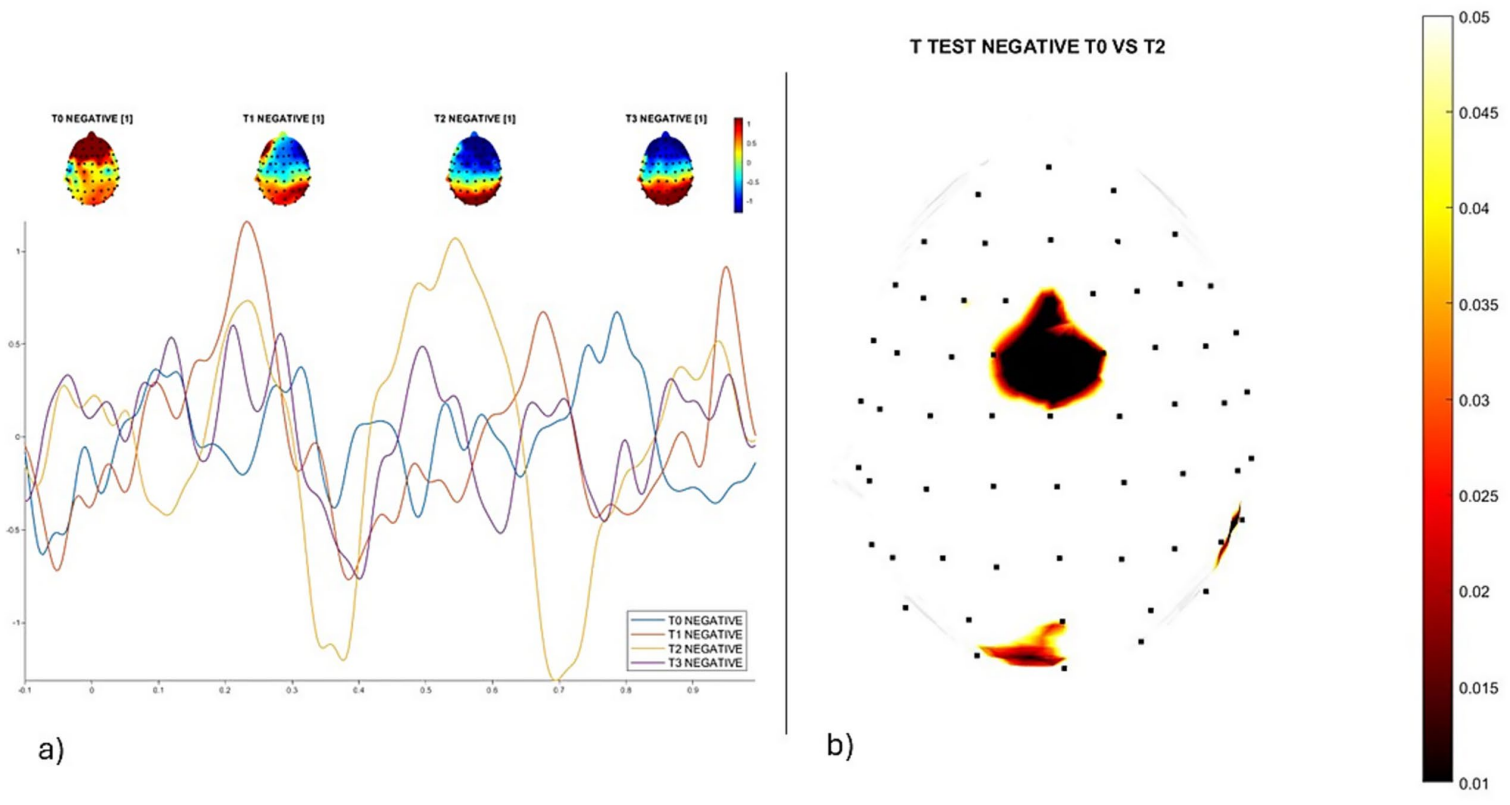
**(a)** Grand-average ERPs elicited by negative stimuli during the emotional Stroop task across sessions with corresponding scalp topographies. **(b)** Topographic representation of electrode-wise statistical results from cluster-based permutation *t*-tests comparing ERP responses to negatively valenced stimuli between T0 and T2.

The delayed 200-related activity showed a session-dependent modulation, with increased amplitude following active stimulation (T2) and smaller amplitude changes at sham (T1) and follow-up (T3) (Figure 6a). Paired-sample comparisons indicated a significant difference between T0 and T2, with a significant cluster over frontocentral midline electrodes and occipital and right inferior-temporal regions (Figure 6b). Latencies of main ERPs were similar between the different sessions.

## 4 Discussion

The present study investigated behavioral and electrophysiological correlates of accelerated intermittent theta-burst stimulation (iTBS) applied to the dorsolateral prefrontal cortex in Huntington’s disease, using a within-subject, fixed-order design that included a sham exposure phase.

The following paragraphs report detailed comments on main results.

### 4.1 Behavioral and cognitive changes

Behavioral and cognitive changes were selectively observed following active stimulation and were absent during the sham condition. Improvements were confined to affective, executive, and social-cognitive domains, whereas other cognitive and clinical indices remained largely unchanged. This dissociation supports a domain-specific pattern of association and suggests that the accelerated delivery of iTBS, through increased temporal density of stimulation sessions, may preferentially engage prefrontal networks supporting higher-order cognitive control, emotional regulation, and socio-emotional integration (Caillaud et al., 2024). In HD, early disruption of frontostriatal and frontocortical networks disproportionately affects these domains (Rosas et al., 2002; Larsen et al., 2016; Crocker and Heller, 2010), rendering them plausible targets for intensive prefrontal neuromodulation.

Previous studies have reported that rTMS can modulate neuropsychiatric symptoms in neurodegenerative conditions, although findings have been heterogeneous, likely reflecting variability in stimulation parameters, cortical targets, and outcome measures (Liu et al., 2024). Within this context, the present findings suggest that use of an accelerated protocol may increase sensitivity to detect domain-specific effects, particularly in disorders characterized by uneven network vulnerability, such as HD. Consistent with this interpretation, iTBS was associated with greater modulation of cognitive–affective domains than with diffuse behavioral changes.

Cognitive outcomes further supported this interpretation. Executive functioning and social cognition—both reliant on prefrontal-mediated integration of internal states, external cues, and goal-directed behavior—were associated with improvement following active stimulation and showed partial retention at follow-up. In contrast, processing speed exhibited only modest and inconsistent changes, supporting the interpretation that accelerated iTBS over the DLPFC preferentially influences higher-order integrative processes rather than elementary cognitive operations.

#### 4.1.1 Event related potentials-Stroop test

Electrophysiological findings provided converging, albeit indirect, mechanistic insight. At baseline, event-related potentials (ERPs) were markedly abnormal, with evident waveform dispersion and latency delays, consistent with profound frontal network dysfunction. The processes indexed by the N200 and N450 components—associated with conflict monitoring and response selection, semantic integration, and later attentional control (Folstein and Van Petten, 2008; Kutas and Federmeier, 2011)—were substantially altered even in patients who were not in advanced disease stages. The Stroop paradigm, particularly in its emotional variant, proved sensitive to these alterations.

Following active iTBS, ERP findings indicated a mild modulation of residual network engagement. Specifically, increases in N200 amplitude were observed without normalization of latency, suggesting enhanced recruitment or synchronization of prefrontal networks rather than recovery of intact processing dynamics. This pattern is consistent with a domain-specific effect and may reflect the capacity of accelerated stimulation schedules to amplify residual network responsiveness transiently. ERP modulation was most evident during the emotional Stroop task and in response to negatively valenced stimuli, which are particularly relevant to social-cognitive and affective dysfunction in HD (Zarotti et al., 2026).

Importantly, the persistence of electrophysiological abnormalities despite measurable behavioral improvement underscores that clinical gains may occur in the absence of full neurophysiological normalization, a dissociation widely documented in neuromodulation and biomarker research (Leuchter et al., 2012). In this context, ERP measures may reflect trait-like vulnerability of frontal networks rather than state-dependent markers of clinical response.

### 4.2 Effects on motor symptoms

The present protocol was primarily aimed at addressing cognitive and affective dysfunction in early Huntington’s disease, selecting the dorsolateral prefrontal cortex as the cortical target rather than the motor or supplementary motor cortex, although intervention in executive and social-cognitive domains could also provide an overall benefit to motor performance.

In our patients no significant changes were observed in motor measures (UHDRS), despite selective improvements in several prefrontal functions. This dissociation is consistent with the known pathophysiology of Huntington’s disease, in which cognitive and affective disturbances may emerge early and follow partially independent trajectories relative to motor impairment (Paulsen et al., 2008), with a specific influence on vulnerable prefrontal networks, in line with models of functionally segregated frontostriatal circuits (Snowden et al., 2003; Alexander et al., 1986).

These findings are in line with models of distributed network dysfunction in Huntington’s disease, where distinct functional domains may show differential sensitivity to neuromodulatory interventions depending on network vulnerability and disease stage.

#### 4.2.1 Consideration of the stimulation protocol

A key methodological aspect of this study is the use of a fixed-order design. Although counterbalanced randomized designs are generally regarded as the gold standard, their use in accelerated neuromodulation paradigms presents significant challenges. Accelerated stimulation protocols may produce cumulative and temporally extended effects, which can influence subsequent conditions even after short washout intervals (Rossi et al., 2009).

The absence of significant changes following the sham procedure, along with the domain-specific rather than generalized effect after active stimulation, supports the validity of the protocol. This approach may represent a compromise between traditional internal validity and biological plausibility, prioritizing the latter to minimize carry-over confounds.

### 4.3 Study limitations

Several limitations should be acknowledged. The absence of randomization and operator blinding limits causal inference and leaves open the possibility of expectation or experimenter-related effects. However, the lack of change during the sham phase and the selective nature of post-stimulation effects reduce the likelihood that the observed findings are solely attributable to non-specific factors. These considerations are particularly relevant in the context of rare neurodegenerative diseases such as Huntington’s disease, where small sample sizes, inter-individual variability, and feasibility constraints often necessitate adapted experimental designs. In such settings, within-subject longitudinal approaches may offer increased sensitivity to detect signal, especially in early-phase or proof-of-concept studies.

In addition, no direct measures of functional or structural connectivity were acquired, preventing specific conclusions regarding the involvement of striatal components of frontostriatal networks. The interpretation of network-level effects should therefore be considered indirect and hypothesis-generating. Finally, the small sample size limits generalizability, and findings should be interpreted as exploratory.

Future studies should aim to integrate biologically informed control conditions, potentially combining sham exposure with alternative active control targets or parametric modulation of stimulation timing, in order to better disentangle specificity of effects while accounting for the cumulative properties of accelerated protocols.

### 4.4 Final remarks

This sham-controlled pilot study suggests that an accelerated iTBS protocol targeting the left dorsolateral prefrontal cortex may be associated with domain-specific neuromodulatory effects in HD, shaped by the interaction between stimulation timing, cortical target, and functional domain. Rather than producing generalized cognitive or behavioral changes, the applied protocol was associated with selective modulation of affective, executive, and social-cognitive functions.

Although exploratory and limited by sample size, these findings underscore the relevance of protocol design—particularly accelerated stimulation schedules—in influencing the functional specificity of neuromodulatory outcomes. Future work should therefore focus on how protocol characteristics—such as acceleration, session density, and temporal spacing—interact with specific functional domains, particularly executive function and social cognition, rather than on global efficacy alone, to determine the durability and clinical relevance of neuromodulatory effects in HD.

## Data Availability

The raw data supporting the conclusions of this article will be made available by the authors, without undue reservation.
