# Supplementary material for: Accelerated prefrontal intermittent theta-burst stimulation in Huntington’s disease: a within-subject study of domain-specific behavioral and event-related potential changes
Source: Front Hum Neurosci. 2026 Jul 16;20:1891708. doi: 10.3389/fnhum.2026.1891708 (PMC13422222; doi:10.3389/fnhum.2026.1891708)
Supplement: Supplementary file 1 [file Table_1.DOCX]

Supplementary details to 4.2

Depressive symptoms showed a small, non-significant reduction (BDI-II Δ = −0.9, p = 0.43), while apathy scores increased slightly without statistical significance (AES Δ = +0.8, p = 0.21). Executive performance remained stable, with no significant changes in processing speed (SDMT Δ = −1.1, p = 0.17) or cognitive flexibility. Social-cognitive performance was also unchanged (FACE Δ = +0.3, p = 1.00).

Supplementary Table 1: Normality of distributions of cognitive variable - Shapiro–Wilk test

| **Variable** | **Time Point** | **Shapiro-Wilk Stat** | **p-value** | **Normality** |
| --- | --- | --- | --- | --- |
| **SDMT** | T0 | 0.9095 | 0.2774 | Yes |
| **SDMT** | T1 | 0.8949 | 0.1923 | Yes |
| **SDMT** | T2 | 0.9136 | 0.3065 | Yes |
| **SDMT** | T3 | 0.9354 | 0.5033 | Yes |
| **TRAIL-B** | T0 | 0.878 | 0.1238 | Yes |
| **TRAIL-B** | T1 | 0.8754 | 0.1154 | Yes |
| **TRAIL-B** | T2 | 0.8798 | 0.13 | Yes |
| **TRAIL-B** | T3 | 0.8983 | 0.2098 | Yes |
| **DEX** | T0 | 0.9735 | 0.9213 | Yes |
| **DEX** | T1 | 0.9721 | 0.9094 | Yes |
| **DEX** | T2 | 0.9565 | 0.7458 | Yes |
| **DEX** | T3 | 0.9734 | 0.9202 | Yes |
| **SET** | T0 | 0.9337 | 0.4849 | Yes |
| **SET** | T1 | 0.9367 | 0.517 | Yes |
| **SET** | T2 | 0.9597 | 0.7823 | Yes |
| **SET** | T3 | 0.9023 | 0.2324 | Yes |
| **FACE** | T0 | 0.9434 | 0.5911 | Yes |
| **FACE** | T1 | 0.9524 | 0.6971 | Yes |
| **FACE** | T2 | 0.9504 | 0.6735 | Yes |
| **FACE** | T3 | 0.9275 | 0.4239 | Yes |
| **EKMAN 60f** | T0 | 0.8945 | 0.1906 | Yes |
| **EKMAN 60f** | T1 | 0.9195 | 0.3525 | Yes |
| **EKMAN 60f** | T2 | 0.9316 | 0.4637 | Yes |
| **EKMAN 60f** | T3 | 0.8858 | 0.1519 | Yes |

# Supplementary Table 2 – Paired Comparisons Δ

All variables were analyzed using paired tests (paired t-test or Wilcoxon signed-rank), with Shapiro–Wilk assessment of normality of the difference scores. Effect sizes are reported as Cohen’s dz for parametric tests and r for non-parametric tests. p-values were FDR-corrected (Benjamini–Hochberg).

| Variable | Comparison | Delta | p_FDR | Effect_size | Effect_type | Significance | N |
| --- | --- | --- | --- | --- | --- | --- | --- |
| BACK | T0→T1 | -0.9 | 0.5918 | -0.483 | r | ns | 10 |
| BACK | T1→T2 | -6.1 | 0.0039 | -1.637 | dz | ** | 10 |
| BACK | T2→T3 | 1.4 | 0.0465 | 0.979 | dz | * | 10 |
| BACK | T0→T3 | -5.6 | 0.0037 | -1.694 | dz | ** | 10 |
| AES | T0→T1 | 0.8 | 0.3475 | 0.427 | dz | ns | 10 |
| AES | T1→T2 | -6.1 | 0.0026 | -2.010 | dz | ** | 10 |
| AES | T2→T3 | -1.1 | 0.8629 | -0.500 | r | ns | 10 |
| AES | T0→T3 | -6.4 | 0.0098 | -0.886 | r | ** | 10 |
| SET | T0→T1 | -0.2 | 0.4533 | -0.806 | r | ns | 10 |
| SET | T1→T2 | 0.5 | 0.1915 | 0.588 | dz | ns | 10 |
| SET | T2→T3 | -0.9 | 0.0784 | -0.818 | dz | ns | 10 |
| SET | T0→T3 | -0.6 | 0.3475 | -0.420 | dz | ns | 10 |
| FACE | T0→T1 | 0.3 | 1.0000 | -0.161 | r | ns | 10 |
| FACE | T1→T2 | 4.0 | 0.0032 | 1.852 | dz | ** | 10 |
| FACE | T2→T3 | -1.5 | 0.0499 | -0.949 | dz | * | 10 |
| FACE | T0→T3 | 2.8 | 0.0784 | 0.825 | dz | ns | 10 |
| EKMAN | T0→T1 | -2.8 | 0.0269 | -1.107 | dz | * | 10 |
| EKMAN | T1→T2 | 1.4 | 0.2520 | 0.523 | dz | ns | 10 |
| EKMAN | T2→T3 | -0.6 | 0.6958 | -0.209 | dz | ns | 10 |
| EKMAN | T0→T3 | -2.0 | 0.1407 | -0.671 | dz | ns | 10 |
| DEX | T0→T1 | 0.4 | 0.6958 | 0.194 | dz | ns | 10 |
| DEX | T1→T2 | -6.3 | 0.0037 | -1.710 | dz | ** | 10 |
| DEX | T2→T3 | 2.6 | 0.1583 | 0.636 | dz | ns | 10 |
| DEX | T0→T3 | -3.3 | 0.0189 | -1.200 | dz | * | 10 |
| SDMT | T0→T1 | -1.1 | 0.2953 | -0.472 | dz | ns | 10 |
| SDMT | T1→T2 | 2.9 | 0.0673 | 0.875 | dz | ns | 10 |
| SDMT | T2→T3 | -1.7 | 0.2953 | -0.473 | dz | ns | 10 |
| SDMT | T0→T3 | 0.1 | 0.9916 | 0.024 | dz | ns | 10 |
| TRAIL_B | T0→T1 | 1.0 | 0.8894 | 0.082 | dz | ns | 10 |
| TRAIL_B | T1→T2 | -15.9 | 0.0021 | -2.267 | dz | ** | 10 |
| TRAIL_B | T2→T3 | 0.0 | 0.6958 | -0.226 | r | ns | 10 |
| TRAIL_B | T0→T3 | -14.9 | 0.1359 | -0.699 | dz | ns | 10 |
| CHAIR_TEST_N | T0→T1 | -0.4 | 0.4533 | -0.341 | dz | ns | 10 |
| CHAIR_TEST_N | T1→T2 | 1.5 | 0.0098 | -0.886 | r | ** | 10 |
| CHAIR_TEST_N | T2→T3 | -0.6 | 0.1407 | -0.886 | r | ns | 10 |
| CHAIR_TEST_N | T0→T3 | 0.5 | 0.3759 | 0.394 | dz | ns | 10 |
| TIMEUPANDGO_SEC | T0→T1 | 0.0 | 1.0000 | 0.000 | dz | ns | 10 |
| TIMEUPANDGO_SEC | T1→T2 | -0.4 | 0.8795 | -0.129 | r | ns | 10 |
| TIMEUPANDGO_SEC | T2→T3 | 0.3 | 0.7326 | 0.170 | dz | ns | 10 |
| TIMEUPANDGO_SEC | T0→T3 | -0.1 | 0.8894 | -0.073 | dz | ns | 10 |

# Supplementary Table 3: Mixed-effects models – Time effects and covariates (Age, MMSE, CAG)

This table reports the results of the linear mixed-effects models (LMM) computed for each outcome variable. Each model included Time_point as a categorical fixed effect and the following baseline predictors: Age, MMSE, CAG. A random intercept for Subject was included to account for repeated measures.

After adjustment for age, baseline MMSE, and CAG repeat length, significant improvements at T2 relative to baseline were confirmed for depression (β = −7.00, SE = 1.01, p < 0.001), apathy (β = −5.64, SE = 1.46, p = 0.00012), executive functioning (DEX), social cognition (FACE), and cognitive flexibility (TRAIL-B), with all p-values < 0.01. Processing speed showed a positive but non-significant trend (p = 0.106). At the 60-day follow-up, improvements remained significant for depression (p < 0.001), executive functioning (DEX; p = 0.003), and social cognition (FACE; p = 0.0007), whereas other cognitive measures did not retain statistical significance

| Outcome | Model summary (Time + covariates) |
| --- | --- |
| BACK | Covariates:  Age: β=0.12, p=0.617 ns, 95% CI [-0.36, 0.61]  MMSE: β=-0.42, p=0.841 ns, 95% CI [-4.53, 3.69]  CAG: β=-0.53, p=0.576 ns, 95% CI [-2.37, 1.31] |
| AES | Covariates:  Age: β=-0.23, p=0.074 ns, 95% CI [-0.48, 0.02]  MMSE: β=0.55, p=0.607 ns, 95% CI [-1.56, 2.67]  CAG: β=-1.53, p=0.001 **, 95% CI [-2.48, -0.59] |
| SET | Covariates:  Age: β=-0.10, p=0.020 *, 95% CI [-0.18, -0.02]  MMSE: β=0.89, p=0.010 *, 95% CI [0.21, 1.57]  CAG: β=-0.30, p=0.052 ns, 95% CI [-0.61, 0.00] |
| FACE | Covariates:  Age: β=-0.19, p=0.094 ns, 95% CI [-0.40, 0.03]  MMSE: β=2.66, p=0.004 **, 95% CI [0.83, 4.48]  CAG: β=-0.49, p=0.240 ns, 95% CI [-1.31, 0.33] |
| EKMAN | Covariates:  Age: β=-0.24, p=0.152 ns, 95% CI [-0.56, 0.09]  MMSE: β=4.20, p=0.002 **, 95% CI [1.48, 6.92]  CAG: β=-0.55, p=0.377 ns, 95% CI [-1.77, 0.67] |
| DEX | Covariates:  Age: β=0.50, p=0.100 ns, 95% CI [-0.10, 1.10]  MMSE: β=-8.42, p=0.001 **, 95% CI [-13.46, -3.38]  CAG: β=1.52, p=0.188 ns, 95% CI [-0.74, 3.77] |
| SDMT | Covariates:  Age: β=-0.22, p=0.289 ns, 95% CI [-0.63, 0.19]  MMSE: β=5.99, p=0.001 ***, 95% CI [2.53, 9.45]  CAG: β=0.01, p=0.986 ns, 95% CI [-1.53, 1.56] |
| TRAIL_B | Covariates:  Age: β=2.18, p=0.045 *, 95% CI [0.04, 4.32]  MMSE: β=-11.15, p=0.224 ns, 95% CI [-29.11, 6.82]  CAG: β=0.95, p=0.817 ns, 95% CI [-7.09, 9.00] |
|  |  |
|  |  |

Supplementary Table 4: Exploratory_Regression_Δ

This table reports the results of the Exploratory regression model, which included age, baseline MMSE score, CAG repeat length, and the baseline value of the corresponding outcome variable as predictors.

| **Outcome** | **Δ** | **Intercept** | **Age** | **MMSE** | **CAG** | **Baseline** | **R²** | **N** |
| --- | --- | --- | --- | --- | --- | --- | --- | --- |
| **BACK** | ΔT1–T0 | -0.02710345145705395 | 0.02022113197125689 | -0.17583240572736067 | 0.12055628721918761 | -0.12521771214900732 | 0.26103493494100116 | 10 |
| **BACK** | ΔT2–T1 | 20.88351178304847 | -0.03498745815135781 | -0.1280530687093999 | -0.3817693671079018 | -0.27225500861173596 | 0.477112448750419 | 10 |
| **BACK** | ΔT3–T2 | -7.4070702773812656 | 0.003941598242218761 | 0.4255008505636714 | -0.08233252481520813 | 0.0668719503235891 | 0.42135828350463 | 10 |
| **AES** | ΔT1–T0 | 27.622351110331934 | -0.12498213763876508 | -0.32820370851314895 | -0.2883395767583283 | 0.04149326804400324 | 0.8617506250410802 | 10 |
| **AES** | ΔT2–T1 | -13.77883370101493 | 0.12435631302278916 | -0.4780097046709393 | 0.3352439935755071 | -0.015207491484484404 | 0.3860018466699501 | 10 |
| **AES** | ΔT3–T2 | 9.670911719494352 | -0.1268046391659942 | 0.9570899928247865 | -0.5590701161063225 | -0.4954064649393845 | 0.3364668953139188 | 10 |

| **Outcome** | **Δ** | **Intercept** | **Age** | **MMSE** | **CAG** | **Baseline** | **R²** | **N** |
| --- | --- | --- | --- | --- | --- | --- | --- | --- |
| **DEX** | ΔT1–T0 | -16.299386523577592 | 0.024585972168618107 | 0.4348665525541964 | 0.03011628505710535 | 0.06862720641919338 | 0.2580187369620709 | 10 |
| **DEX** | ΔT2–T1 | -43.20537981371265 | 0.023453447491887616 | 1.073499205874835 | 0.24001653674102436 | -0.09538681056826188 | 0.8648633931010336 | 10 |
| **DEX** | ΔT3–T2 | 34.73987795450847 | 0.09932416455188306 | -1.4738015432272624 | 0.14339955705418778 | -0.12093336304058228 | 0.18694593736899845 | 10 |
| **SDMT** | ΔT1–T0 | -11.051662190245542 | -0.06655655995194996 | 0.8964241143872428 | -0.21297959000351924 | -0.07287844458326163 | 0.2533499902433288 | 10 |
| **SDMT** | ΔT2–T1 | 15.151846744848738 | 0.02739168482018906 | -0.4448439455222644 | -0.03128133100318645 | -0.014335171892589583 | 0.11525869034340752 | 10 |
| **SDMT** | ΔT3–T2 | -25.819429028813676 | -0.07176364973756573 | 0.6623099752557391 | 0.32691150069690156 | -0.2048828854224943 | 0.4748651097470543 | 10 |
| **TRAIL-B** | ΔT1–T0 | -20.350062724223662 | 0.11323238044456536 | 1.0543499190904375 | -0.10711615607582388 | -0.049403792492174865 | 0.06377585600236946 | 10 |
| **TRAIL-B** | ΔT2–T1 | 53.213680615914974 | -0.31132180185619907 | -1.4484735190948221 | -0.23306398219288094 | -0.032049532722822335 | 0.7197557304849489 | 10 |
| **TRAIL-B** | ΔT3–T2 | 110.24835489922471 | -0.6330460211755251 | -0.5126237819245241 | -1.4275360816087945 | -0.022270521612572314 | 0.378846907767705 | 10 |

| **Outcome** | **Δ** | **Intercept** | **Age** | **MMSE** | **CAG** | **Baseline** | **R²** | **N** |
| --- | --- | --- | --- | --- | --- | --- | --- | --- |
| **FACE** | ΔT1–T0 | 2.8320741858478753 | 0.029811031753787392 | -0.6099783534602162 | 0.15173949824497024 | 0.2578703588415972 | 0.12481645034690236 | 10 |
| **FACE** | ΔT2–T1 | -10.803291010596826 | 0.01950046685501449 | -0.07435920324789086 | 0.2559130120005456 | 0.20509252591603103 | 0.4147381327035804 | 10 |
| **FACE** | ΔT3–T2 | 8.453194353994723 | 0.0064213041463286975 | -0.3942964171358829 | 0.04721554104952089 | -0.06718496860604753 | 0.41700628552439023 | 10 |
| **EKMAN 60f** | ΔT1–T0 | 1.6228405357811049 | -0.0727407263110051 | 0.3387402203743054 | -0.23064534539021694 | 0.004057925156025315 | 0.2044704364450498 | 10 |
| **EKMAN 60f** | ΔT2–T1 | 23.710748215126916 | 0.05574493770053234 | -1.1965302636306303 | 0.06771981870572756 | 0.1109812591354959 | 0.26382522277077247 | 10 |
| **EKMAN 60f** | ΔT3–T2 | -22.844072418240735 | -0.08548546214739187 | 1.806219322749962 | -0.24336210558343105 | -0.2964849467033575 | 0.576153000176911 | 10 |

Supplementary Table 5: Group-level comparisons of correct responses, errors, and omissions in the Stroop task between individuals with Huntington’s disease and healthy controls at baseline (T0).

Independent samples t-tests with Welch correction were used.

Values are reported as group means, t-statistics, and p-values.

|  | **HD mean** | **CG mean** | **t** | **p** |
| --- | --- | --- | --- | --- |
| **STROOP mean-RT** | 2684.9 | 1219.0 | 1.9031430306924242 | 0.08745993410277807 |
| **STROOP n.corr** | 51.7 | 58.0 | -2.0285089400236282 | 0.06871313179956838 |
| **STROOP n.wrong** | 3.7 | 1.6 | 1.3731389910843348 | 0.1883933430311633 |
| **STROOP n.miss** | 4.6 | 0.4 | 1.8808065171835757 | 0.09148163609496468 |
| **E-STROOP mean-RT** | 1722.5 | 1058.8 | 2.970689367492204 | 0.010828381296594768 |
| **E-STROOP n. corr** | 43.8 | 46.6 | -1.5989126740164525 | 0.14277477896649854 |
| **E-STROOP n.wrong** | 0.8 | 0.9 | -0.22056438662814223 | 0.828457508257137 |
| **E-STROOP n.miss** | 3.4 | 0.4 | 1.95099743142927 | 0.08153060824028614 |

|  | N200 latency-  congruent (msec) | N200 latency incongruent | N200 amplitude congruent (uV) | N200 amplitude incongruent  (uV) | N200 amplitude negative  (uV) | N200 amplitude positive  (uV) | N200 latency positive  (msec) | N200 latency negative  (msec) |
| --- | --- | --- | --- | --- | --- | --- | --- | --- |
| patients | 390  (95) | 360 (86) | 0.9  (0.87) | 0.5  (0.6) | 0.5  (0.5) | 0.4  0.5 | 385  (95) | 375  (90) |
| controls | 230  (87) | 210  (65) | 2.8  (1.2) | 1.8  (0.9) | 1.2  (0.5) | 1.1  0.4 | 220  (89) | 211  (76) |
| t test p | <0.0001 | <0.0001 | <0.01 | <0.01 | <0.05 | <0.05 | <0.001 | <0.001 |
|  | N450  latency-  congruent (msec) | N450 latency incongruent | N450 amplitude congruent (uV) | N450 amplitude incongruent  (uV) | N450 amplitude negative  (uV) | N450 amplitude positive  (uV) | N450 latency positive  (msec) | N450 latency negative  (msec) |
| patients | 700  (93) | 710  (88) | 2  (0.9) | 1.8  (0.8) | 0.8  (1) | 0.6  (0.7) | 690  (100) | 780  (98) |
| controls | 480  (98) | 490  (89) | 3.9  (1.2) | 2.8  (0.9) | 4.6  (0.7) | 4  (0.8) | 490  (78) | 510  (81) |
| t test p | <0.001 | <0.001 | <0.05 | <0.05 | <0.01 | <0.01 | <0.001 | <0.001 |

Supplementary Table 6

|  | N200 latency-  congruent (msec) | N200 latency incongruent | N 200 amplitude congruent (uV) | N200 amplitude incongruent  (uV) | N200 amplitude negative  (uV) | N200 amplitude positive  (uV) | N200 latency positive  (msec) | N200 latency negative  (msec) |  |
| --- | --- | --- | --- | --- | --- | --- | --- | --- | --- |
| patients | 390  (95) | 360 (86) | 0.9  (0.87) | 0.5  (0.6) | 0.5  (0.5) | 0.4  0.5 | 385  (95) | 375  (90) |  |
| controls | 230  (87) | 210  (65) | 2.8  (1.2) | 1.8  (0.9) | 1.2  (0.5) | 1.1  0.4 | 220  (89) | 211  (76) |  |
| t test p | <0.0001 | <0.0001 | <0.01 | <0.01 | <0.05 | <0.05 | <0.001 | <0.001 |  |

Mean and standard deviations of amplitude and latencies of N200 and N450 waves in HD patients and controls. Values averaged over Fz-Cz electrodes are shown.

Mean and standard deviations of N200 latency and amplitude in Hd patients and controls

Results of student’s t test are reported.

|  | N450  latency-  congruent (msec) | N450 latency incongruent | N450 amplitude congruent (uV) | N450 amplitude incongruent  (uV) | N450 amplitude negative  (uV) | N450 amplitude positive  (uV) | N450 latency positive  (msec) | N450 latency negative  (msec) |
| --- | --- | --- | --- | --- | --- | --- | --- | --- |
| Patients | 700  93 | 710  88 | 2  0.9 | 1.8  0.8 | 0.8  1 | 0.6  0.7 | 690  100 | 780  98 |
| Controls | 480  98 | 490  89 | 3.9  1.2 | 2.8  0.9 | 4.6  0.7 | 4  0.8 | 490  78 | 510  81 |
| t test p | <0.001 | <0.001 | <0.05 | <0.05 | <0.01 | <0.01 | <0.001 | <0.001 |
